# Guanosine tetraphosphate relieves the negative regulation of *Salmonella* pathogenicity island-2 gene transcription exerted by the AT-rich *ssrA* discriminator region

**DOI:** 10.1038/s41598-018-27780-9

**Published:** 2018-06-21

**Authors:** Timothy Tapscott, Ju-Sim Kim, Matthew A. Crawford, Liam Fitzsimmons, Lin Liu, Jessica Jones-Carson, Andrés Vázquez-Torres

**Affiliations:** 1 Molecular Biology Program, University of Colorado School of Medicine, Aurora, CO USA; 2 Department of Immunology and Microbiology, University of Colorado School of Medicine, Aurora, CO USA; 3 Division of Infectious Diseases, University of Colorado School of Medicine, Aurora, CO USA; 4 Veterans Affairs Eastern Colorado Health Care System, Denver, CO USA

## Abstract

The repressive activity of ancestral histone-like proteins helps integrate transcription of foreign genes with discrepant AT content into existing regulatory networks. Our investigations indicate that the AT-rich discriminator region located between the −10 promoter element and the transcription start site of the regulatory gene *ssrA* plays a distinct role in the balanced expression of the *Salmonella* pathogenicity island-2 (SPI2) type III secretion system. The RNA polymerase-binding protein DksA activates the *ssrAB* regulon post-transcriptionally, whereas the alarmone guanosine tetraphosphate (ppGpp) relieves the negative regulation imposed by the AT-rich *ssrA* discriminator region. An increase in the GC-content of the *ssrA* discriminator region enhances *ssrAB* transcription and SsrB translation, thus activating the expression of downstream SPI2 genes. A *Salmonella* strain expressing a GC-rich *ssrA* discriminator region is attenuated in mice and grows poorly intracellularly. The combined actions of ppGpp and DksA on SPI2 expression enable *Salmonella* to grow intracellularly, and cause disease in a murine model of infection. Collectively, these findings indicate that (p)ppGpp relieves the negative regulation associated with the AT-rich discriminator region in the promoter of the horizontally-acquired *ssrA* gene, whereas DksA activates *ssrB* gene expression post-transcriptionally. The combined effects of (p)ppGpp and DksA on the *ssrAB* locus facilitate a balanced SPI2 virulence gene transcription that is essential for *Salmonella* pathogenesis.

## Introduction

Nontyphoidal *Salmonella enterica* serovar Typhimurium is a common cause of gastroenteritis in immunocompetent individuals and a life-threatening disseminated complication in immunocompromised hosts unable to mount CD4^+^ T cell immunity or IFNγ host responses^1,2^. This intracellular pathogen replicates within *Salmonella*-containing vacuoles (SCV) of epithelial and phagocytic cells in part due to the activity of a type III secretion system that is encoded within the horizontally-acquired *Salmonella* pathogenicity island-2 (SPI2)^3–5^. Effector proteins translocated through the SPI2 type III secretion system minimize contact of SCV with lysosomes and cell host vesicles harboring NADPH phagocyte oxidase or inducible nitric oxide synthase (iNOS)^6–10^. By redirecting SCVs to the trans-Golgi network and exocytic pathway, the SPI2 type III secretion system also aids *Salmonella* in overcoming the nutritional restrictions found in vesicles of the degradative pathway^11,12^.

*Salmonella* initiate SPI2 gene transcription as the transforming SCV microenvironment acidifies and becomes limiting for iron and other divalent cations^13–16^. These signals activate the EnvZ and PhoQ sensor kinases, which catalyze phosphotransfer reactions to their cognate response regulators OmpR and PhoP, respectively^17,18^. PhoP competes with histone-like proteins for binding to the *ssrA* promoter, counter-silencing the repressive activity of these nucleoid-structuring proteins^19,20^. The sensor kinase encoded by the *ssrA* gene senses acidification via several histidine residues in the periplasmic domain^21^. Activated SsrA phosphorylates its cognate SsrB response regulator, which in turn recruits the RNA polymerase to SPI2 genes encoding components of the secretion apparatus, chaperones, and effectors^5,22^. The negative regulation of SPI2 genes is also an important aspect in *Salmonella* pathogenesis. For example, EIIA^Ntr^, which prevents binding of SsrB to promoters of SPI2 target genes, is required for *Salmonella* virulence^23^. Also, the inactivation of SsrB via oxidation or S-nitrosation of Cys^203^ contributes to *Salmonella* virulence^24^. Moreover, binding of the histone-like proteins H-NS and YdgT to AT-rich SPI2 genes represses SPI2 transcription during non-inducing conditions^25,26^. The absence of these histone-like proteins attenuates *Salmonella* in spite of SPI2 overexpression^23,24,26^.

The AT-rich composition of the discriminator region, which is located between the −10 element and transcriptional start site, could be an additional repressive element to transcription of horizontally-acquired genes. Promoters with AT-rich discriminator regions often produce stable, long-lived, open complexes that become saturated with RNA polymerase, aborting initiation of transcription^27,28^. The stringent response is controlled by the RNA polymerase-binding protein DksA and the nucleotide alarmones guanosine tetra/pentaphosphate [(p)ppGpp] that are synthesized in *Salmonella* by the RelA and SpoT proteins. DksA binds to the secondary channel of the RNA polymerase, whereas two molecules of (p)ppGpp bind between the ω and β’ subunits and at the interface of RNA polymerase and DksA^29,30^. DksA and (p)ppGpp exert transcriptional regulation by reducing the half-life of RNA polymerase-DNA open complexes^27,28,31,32^. The stringent response generally activates or represses gene transcription from promoters with AT- or GC-rich discriminator regions, respectively^27,28,31,32^. The preservation of AT-rich discriminator regions in horizontally-acquired genes suggests that the negative control associated with AT-rich discriminators provides a selective advantage to bacterial pathogens.

Microarrays and differential RNA sequencing indicate that the stringent response regulators DksA and (p)ppGpp are required for the activation of SPI2 gene transcription^33–38^. However, the mechanism by which DksA and (p)ppGpp regulate expression of the SPI2 virulence program remains unknown. Herein, we tested the hypothesis that the stringent response regulators DksA and (p)ppGpp contribute to *Salmonella* virulence by relieving the negative regulation imposed by the AT-rich discriminator region of the *ssrAB* locus encoding the SPI2 master two-component regulatory system.

## Results

### DksA and (p)ppGpp promote intracellular replication of *Salmonella*

**Figure 1:**
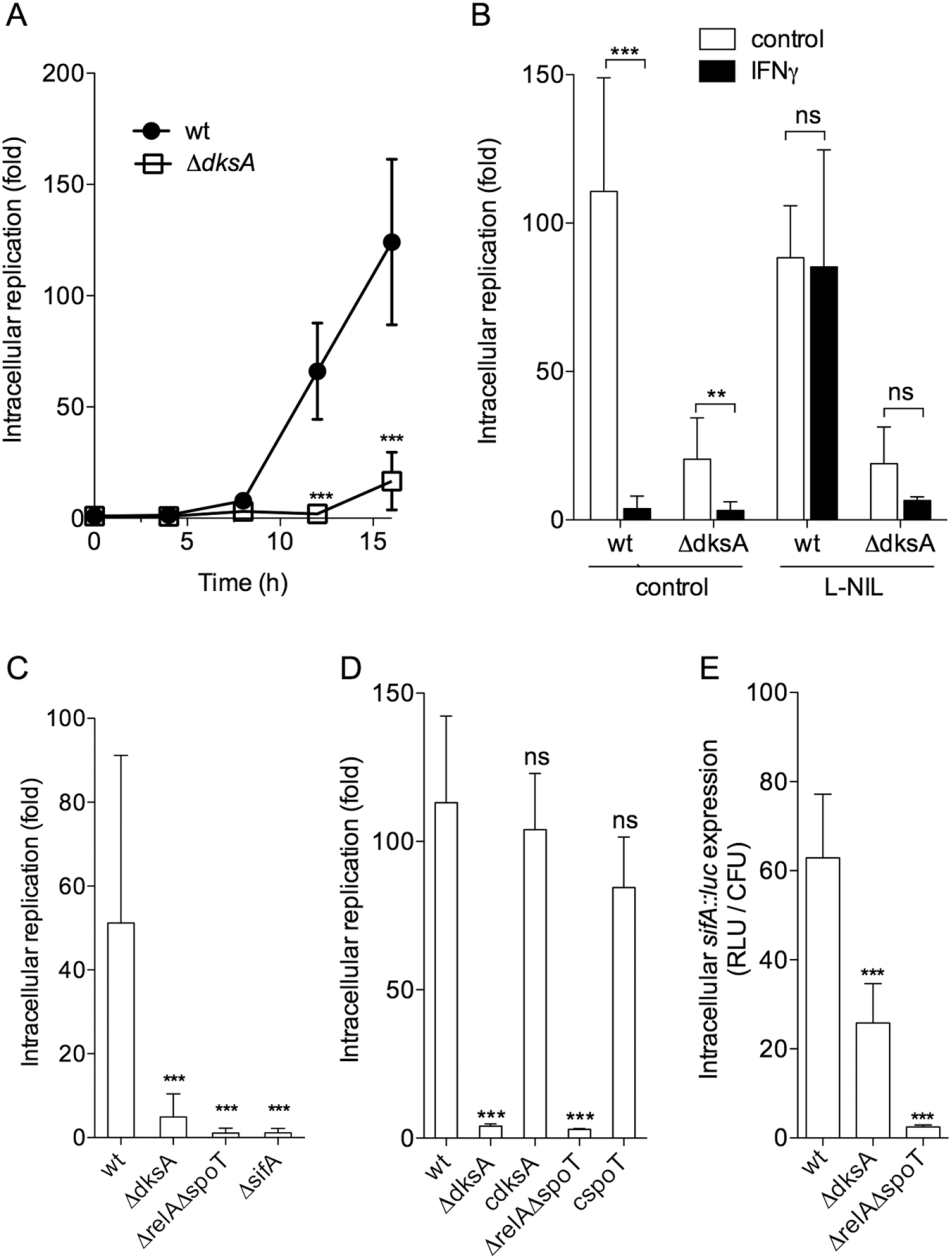
Contributions of DksA and (p)ppGpp to the ability of *Salmonella* to replicate intracellularly. Intracellular replication of wild-type (wt) and mutant *Salmonella* was quantified over time (**A**) or 18 h post-infection (**B**–**D**) in J774A.1 macrophage-like cells. Where indicated, J774A.1 cells were stimulated with 200 U/ml IFNγ 24 h prior to infection, or treated since the time of infection with 960 μM of the selective iNOS inhibitor L-NIL. Intracellular expression of *sifA::luc* 8 h after J774A.1 cells were infected with *Salmonella* (**E**). Non-significant (ns), *p < 0.05, **p < 0.01, or ***p < 0.001 compared to wild-type controls. The data represent the mean ± S.D. from 3–19 biological replicates.

By regulating the expression of gene products that maintain NADPH/NADP^+^ and GSH/GSSG redox homeostasis, DksA and (p)ppGpp protect *Salmonella* against the antimicrobial activity associated with NADPH phagocyte oxidase and iNOS hemoproteins^39–43^. To examine whether DksA and (p)ppGpp play additional roles during the intracellular growth of *Salmonella*, we measured the replication of Δ*dksA* and Δ*relA* Δ*spoT Salmonella* in J774A.1 macrophage-like cells. Wild-type *Salmonella* began to replicate 8 h post-infection, reaching over 100-fold higher bacterial burdens 16 h after the initial infection (Fig. 1A). In contrast, Δ*dksA Salmonella* grew poorly (Fig. 1A). Under the experimental conditions tested, our J774A.1 cells do not generate detectable amounts of reactive oxygen species in response to *Salmonella*^43^. These findings suggest that the growth defect of Δ*dksA Salmonella* in this population of J774A.1 cells cannot be attributed to its reported hypersusceptibility to oxidative stress^39–43^. Treatment of J774A.1 cells with IFNγ arrested growth of both wild-type and Δ*dksA Salmonella* (Fig. 1B). Wild-type bacteria, but not the Δ*dksA* mutant, grew in IFNγ-activated macrophages treated with the iNOS specific inhibitor N-iminoethyl-L-lysine (L-NIL) (Fig. 1B). As expected, L-NIL inhibited NO synthesis (Fig. S1A). Together, these investigations suggest that DksA can aid in the intracellular replication of *Salmonella* independently of its promotion of antioxidant and antinitrosative defenses.

Since DksA and (p)ppGpp often coregulate the RNA polymerase, we also tested the intracellular growth of a Δ*relA* Δ*spoT* strain. A Δ*relA* Δ*spoT Salmonella* strain exhibited profound intracellular growth defects (Fig. 1C). The failure of Δ*dksA* and Δ*relA* Δ*spoT Salmonella* strains to replicate intracellularly resembles phenotypes reported for strains deficient in SPI2 genes^3–5^. Accordingly, an isogenic strain lacking the SPI2 effector *sifA*, whose product is necessary for maintaining integrity of the SCV^44^, grew as poorly in J774A.1 cells as Δ*dksA* and Δ*relA* Δ*spoT Salmonella* controls (Fig. 1C). The growth defect of Δ*dksA* and Δ*relA* Δ*spoT Salmonella* could be complemented by *dksA* and *spoT* alleles expressed in the chromosome (Fig. 1D). We next tested whether the stringent response regulators DksA and (p)ppGpp contribute to the intracellular expression of *sifA*. Compared to wild-type controls, both Δ*dksA* and Δ*relA* Δ*spoT Salmonella* expressed low levels of the SPI2 effector *sifA* in J774A.1 macrophage-like cells (Fig. 1E). As shown previously^33–35,37,38^, wild-type *Salmonella* grown for 3 h in 8 μM MgCl_2_ N9 medium expressed all SPI2 promoters tested; however, Δ*dksA* or Δ*relA* Δ*spoT Salmonella* did not stimulate expression of any SPI2 genes examined (Fig. S1B).

Collectively, these observations raise the possibility that the stringent response regulators DksA and (p)ppGpp help *Salmonella* grow in macrophages by controlling the expression of the SPI2 type III secretion system.

### Contributions of DksA, (p)ppGpp, and SsrB to *Salmonella* pathogenesis

**Figure 2:**
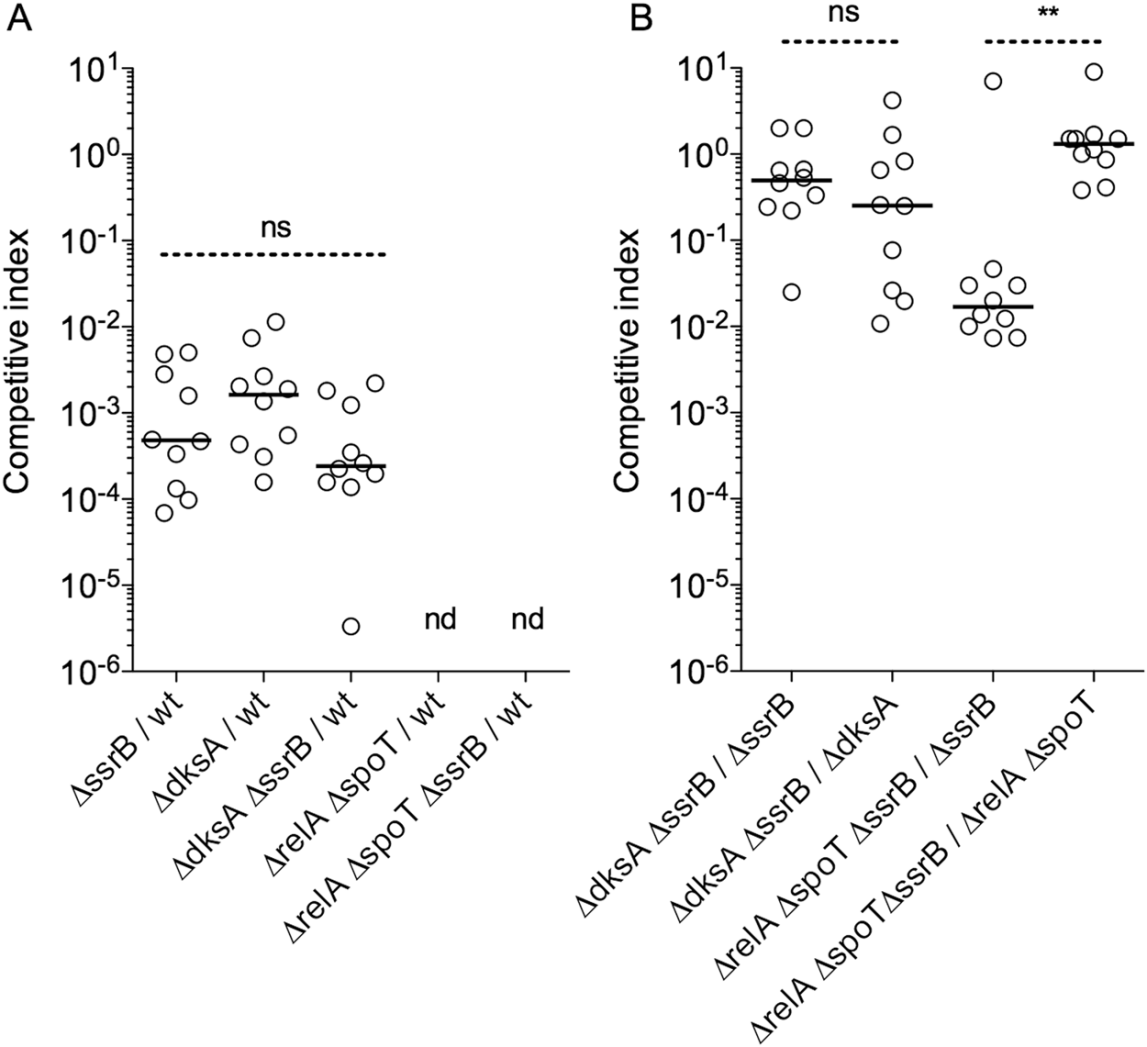
Codependence of SsrB, ppGpp, and DksA in *Salmonella* pathogenesis. Competitive indexes of *Salmonella* strains recovered from spleens of C57BL/6 mice 3 days after infection. Mice were inoculated intraperitoneally with 10^2^ (**A**) or 10^5^ (**B**) CFU of the indicated *Salmonella* strains. No detectable (nd) CFU were isolated for the Δ*relA* Δ*spoT* strain under the experimental conditions used in panel A. Competitive index was determined according to the equation: (strain 1/strain 2)_output_/(strain 1/strain 2)_input_. Non-significant (ns), **p < 0.01.

Since DksA and (p)ppGpp play broad roles in gene transcription^33,38,39,45,46^, we deemed it important to quantify the extent that these stringent response regulators rely on the SPI2 type III secretion system to promote *Salmonella* pathogenesis. When compared to wild-type controls, the number of Δ*ssrB*, Δ*dksA, or* Δ*dksA* Δ*ssrB Salmonella* was about 1,000-fold lower in spleens (Fig. 2A) and livers (Fig. S2A) of C57BL/6 mice. Strains unable to generate (p)ppGpp were more attenuated than Δ*ssrB* or Δ*dksA Salmonella*, as demonstrated by their complete elimination from spleens and livers 3 days after intraperitoneal inoculation (Figs 2A and S2A). To determine fitness of Δ*relA* Δ*spoT Salmonella*, C57BL/6 mice were inoculated with 10^5^ CFU of each Δ*relA* Δ*spoT* and Δ*relA* Δ*spoT* Δ*ssrB Salmonella*. The Δ*relA* Δ*spoT* Δ*ssrB* mutant had a competitive index of ~1 when compared to Δ*relA* Δ*spoT Salmonella*, but showed a 100-fold lower competitive index than Δ*ssrB Salmonella* (Figs 2B and S2B). These data suggest that (p)ppGpp can participate in *Salmonella* virulence in SPI2-dependent and -independent ways. To better calculate the apparent codependency of SsrB and DksA, we used the method described by Beuzon and Holden to quantify virulence gene interactions *in vivo*^44,47^. Groups of C57BL/6 mice were inoculated with 10^5^ CFU of Δ*ssrB* Δ*dksA* in combination with Δ*ssrB* or Δ*dksA Salmonella*. The Δ*dksA* Δ*ssrB* double mutant strain was isolated from spleen and liver tissue in similar numbers to Δ*ssrB* or Δ*dksA* single mutants (Figs 2B and S2B), suggesting that the role played by this RNA polymerase-binding protein in *Salmonella* pathogenesis appears to be strongly co-dependent on the SPI2 master regulator SsrB.

### Requirement of DksA and (p)ppGpp for the activation of *ssrAB* transcription

**Figure 3:**
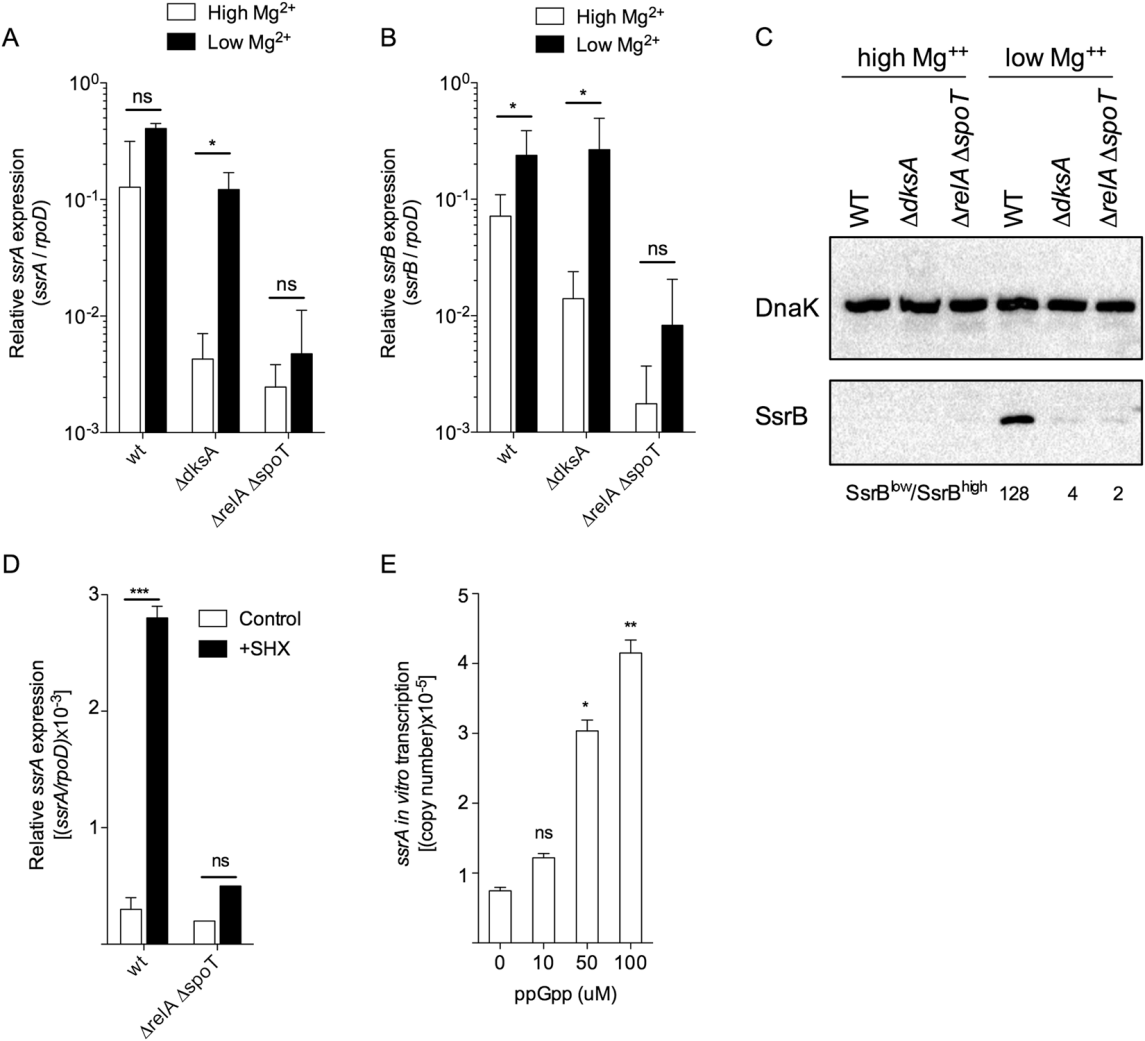
The alarmone ppGpp directly activates *ssrA* gene transcription. The abundance of *ssrA* (**A**) and *ssrB* (**B**) mRNA was quantified by qRT-PCR in *Salmonella* grown for 3 h in 8 μM (low) or 10 mM (high) MgCl_2_ N9 media. The data, which are from 4 biological replicates and are plotted as the mean ± S.D., represent transcripts levels normalized to the housekeeping gene *rpoD*. Western blot of SsrB-FLAG in *Salmonella* grown for 3 h in high or low MgCl_2_ N9 medium (**C**). The blot is representative of 4 biological replicates. The abundance of the DnaK internal control was measured for comparison. The relative amounts of SsrB protein were compared between *Salmonella* grown in low and high Mg^++^ (SsrB^low^/SsrB^high^). The amount of *ssrA* mRNA induced by serine hydroxamate (SHX) was quantified by qRT-PCR in *Salmonella* grown to log-phase in M9 minimal medium (**D**). Data represent mean ± S.D. transcripts levels normalized to the housekeeping gene *rpoD*. Effects of increasing ppGpp concentrations on P*ssrA in vitro* transcription using the pTIM-*ssrA* template (**E**). The data, which are plotted as the mean ± S.D., represent absolute copy number determined by qRT-PCR from 6–8 biological replicates. Non-significant (ns), *p < 0.05, **p < 0.01, or ***p < 0.001 as compared to high Mg^2+^ (**A**,**B**) or untreated (**D**,**E**) controls. An independent, uncropped, blot of panel C can be seen in Fig. S6A.

We examined whether DksA and (p)ppGpp participate in the transcriptional activation of the *ssrA* and *ssrB* genes that encode the master two-component regulatory system that activates SPI2 expression. Wild-type *Salmonella* up-regulated the expression of *ssrA* (Fig. 3A) and *ssrB* mRNA (Fig. 3B) 3 h after culture in 8 μM MgCl_2_ N9 medium. We also observed that Δ*dksA Salmonella* induced excellent *ssrA* and *ssrB* expression upon culture in 8 μM MgCl_2_ N9 medium (Fig. 3A,B). These findings indicate that DksA does not appear to regulate *ssrA* or *ssrB* gene transcription. Since Δ*dksA Salmonella* induced *ssrB* expression but failed to globally activate SPI2 transcription, Western blotting was used to visualize the amount of SsrB protein in wild-type and Δ*dksA Salmonella*. Wild-type *Salmonella* harbored low concentrations of SsrB protein when grown under non-inducing 10 mM MgCl_2_ N9 medium, but harbored high concentrations of this response regulator 3 h after growth in 8 μM MgCl_2_ N9 medium (Fig. 3C). Compared to wild-type controls, Δ*dksA Salmonella* expressed much lower concentrations of SsrB protein upon culture in 8 μM MgCl_2_ N9 medium. Expression of a *dksA* allele reestablished production of SsrB protein in Δ*dksA Salmonella* (Fig. S3A). In view of the abundant *ssrB* mRNA seen in Δ*dksA Salmonella*, deficient production of SsrB protein indicates that DksA may regulate the expression of this response regulator post-transcriptionally.

Compared to Δ*dksA Salmonella* and wild-type controls, Δ*relA* Δ*spoT Salmonella* had significantly lower basal levels of *ssrA* and *ssrB* mRNA in non-inducing 10 mM MgCl_2_ N9 medium (Fig. 3A,B). Growth of Δ*relA* Δ*spoT Salmonella* in 8 μM MgCl_2_ N9 medium did not stimulate *ssrA* or *ssrB* expression. As predicted from these transcriptional profiles, Δ*relA* Δ*spoT Salmonella* contained extremely low amounts of the SsrB protein (Fig. 3C). The lack of *ssrA* and *ssrB* expression in Δ*relA* Δ*spoT Salmonella* raises the possibility that (p)ppGpp may directly activate *ssrAB* gene transcription. To test this idea, serine hydroxamate (SHX) was added to *Salmonella* grown to log phase in M9 minimal media. Addition of SHX to rapidly growing bacteria inhibits seryl-tRNA synthetase; the resulting accumulation of deacylated tRNAs stimulates (p)ppGpp synthesis from RelA^48^. The expression of *ssrA* mRNA was induced after the addition of SHX (Fig. 3D). SHX, however, did not induce *ssrA* transcription in Δ*relA* Δ*spoT Salmonella*. To further examine the possibility that (p)ppGpp directly activates *ssrA* transcription, ppGpp was added to *in vitro* transcription reactions containing the pTIM-*ssrA* plasmid template (Fig. S4). *ssrA* transcripts were quantified by combining *in vitro* transcription reactions with a highly sensitive and specific qRT-PCR method^49,50^. This approach revealed that ppGpp directly stimulates *ssrA in vitro* transcription in a concentration-dependent manner (Fig. 3E). These data indicate that (p)ppGpp suffices to activate *ssrA* transcription in *Salmonella*.

### The *ssrA* AT-rich discriminator region facilitates *Salmonella* virulence

**Figure 4:**
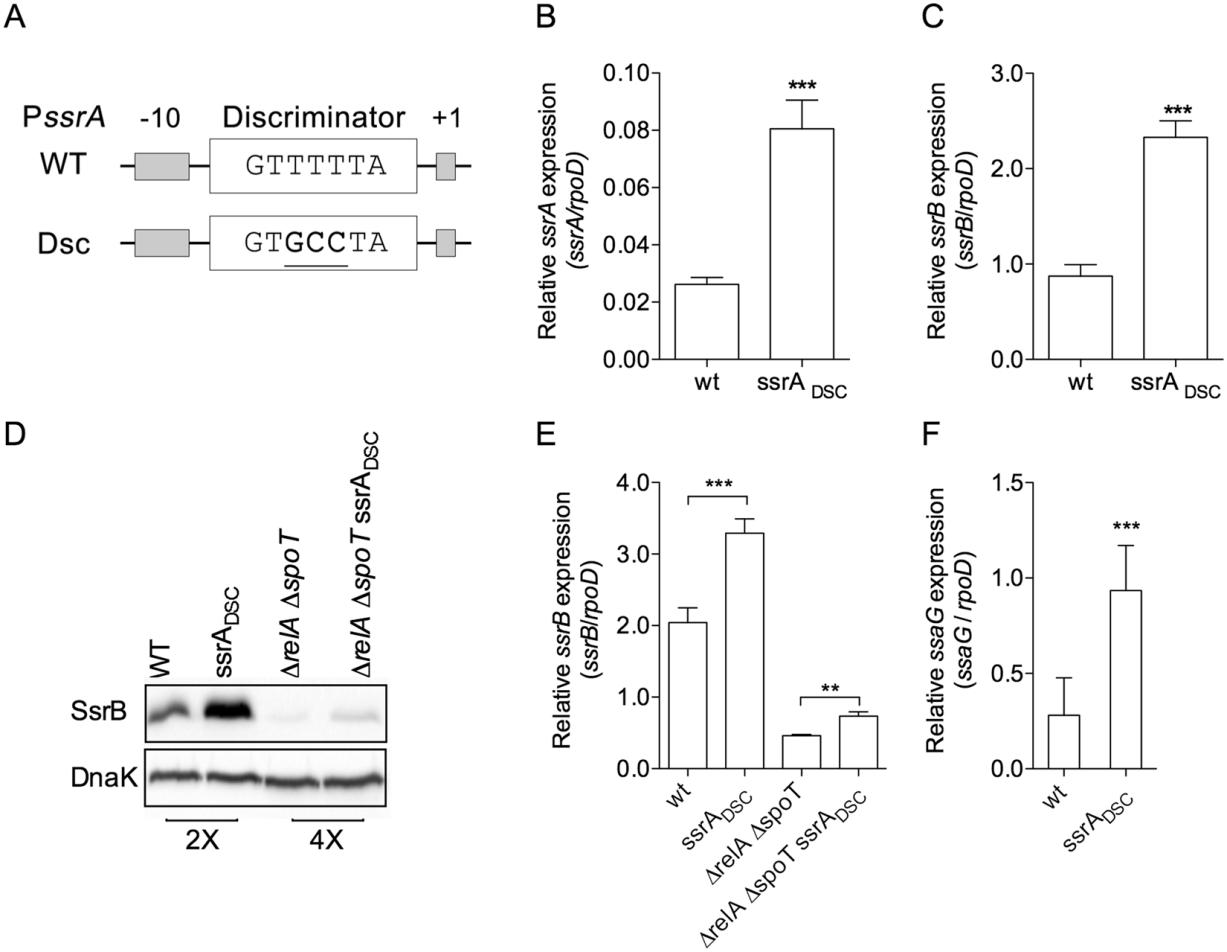
The AT-rich discriminator region of *ssrA* serves as a negative regulator of SPI2 expression. A mutant discriminator region of the *ssrA* promoter was expressed from the native locus in the *Salmonella* chromosome (**A**). The abundance of *ssrA* (**B**), *ssrB* (**C**,**E**) and *ssaG* (**F**) transcripts was measured by qRT-PCR in *Salmonella* grown for the indicated times in LB broth. Data, which are depicted as mean ± S.D. from 6–8 biological replicates, were normalized to the mRNA levels of the *rpoD* housekeeping gene. Western blot analysis the SsrB protein in *Salmonella* grown in LB broth for 5 h (**D**). **p < 0.01; ***p < 0.001. The abundance of the DnaK chaperone was measured as an internal control. Data are representative of 3 independent experiments. An independent, uncropped blot, of panel D can be seen in Fig. S6B.

Since (p)ppGpp often activates gene transcription from AT-rich discriminator regions that form stable, long-lived, open complexes with RNA polymerase^27,28,31^, we focused our attention on the AT-rich P*ssrA* discriminator region. We reasoned that increasing the GC-content would modulate the negative regulation associated with the AT-rich discriminator region of P*ssrA*. To test this model, we engineered three substitutions at the native locus in the *Salmonella* chromosome that increased the GC content in the *ssrA* discriminator region, yielding the *ssrA*_Dsc_ *Salmonella* strain (Figs 4A and S5A). Transcription of *ssrA* (Fig. 4B) and *ssrB* (Fig. 4C) was markedly higher in *ssrA*_Dsc_ *Salmonella* than wild-type controls grown in LB broth to early stationary phase. Consistent with higher levels of *ssrA* and *ssrB* mRNA, the concentration of SsrB protein was higher in *ssrA*_Dsc_ *Salmonella* grown in stationary phase in LB broth than in wild-type isogenic controls (Fig. 4D). The concentration of SsrB was also higher in *ssrA*_Dsc_ *Salmonella* than wild-type controls grown in N9 low Mg^2+^ media (Fig. S5B). Expression of *ssrA*_Dsc_ allele in the Δ*relA* Δ*spoT* background dramatically reduced the amount of intracellular SsrB protein in early stationary phase *Salmonella*, suggesting that the derepression of *ssrAB* transcription associated with a GC-rich *ssrA* discriminatory region is dependent on (p)ppGpp. It should be noted that Δ*relA* Δ*spoT ssrA*_*Dsc*_ *Salmonella* expressed more SsrB than Δ*relA* Δ*spoT* controls (Fig. 4D), but less than wild-type and *ssrA*_*Dsc*_ controls. The concentration of SsrB protein (Fig. 4D) reflected *ssrB* mRNA levels (Fig. 4C,E). Transcription of the SsrB-regulated *ssaG* gene was also upregulated (*p* < 0.001) in *ssrA*_Dsc_ *Salmonella* compared to isogenic wild-type bacteria (Fig. 4F).

### Virulence of *ssrA*_Dsc_*Salmonella*

**Figure 5:**
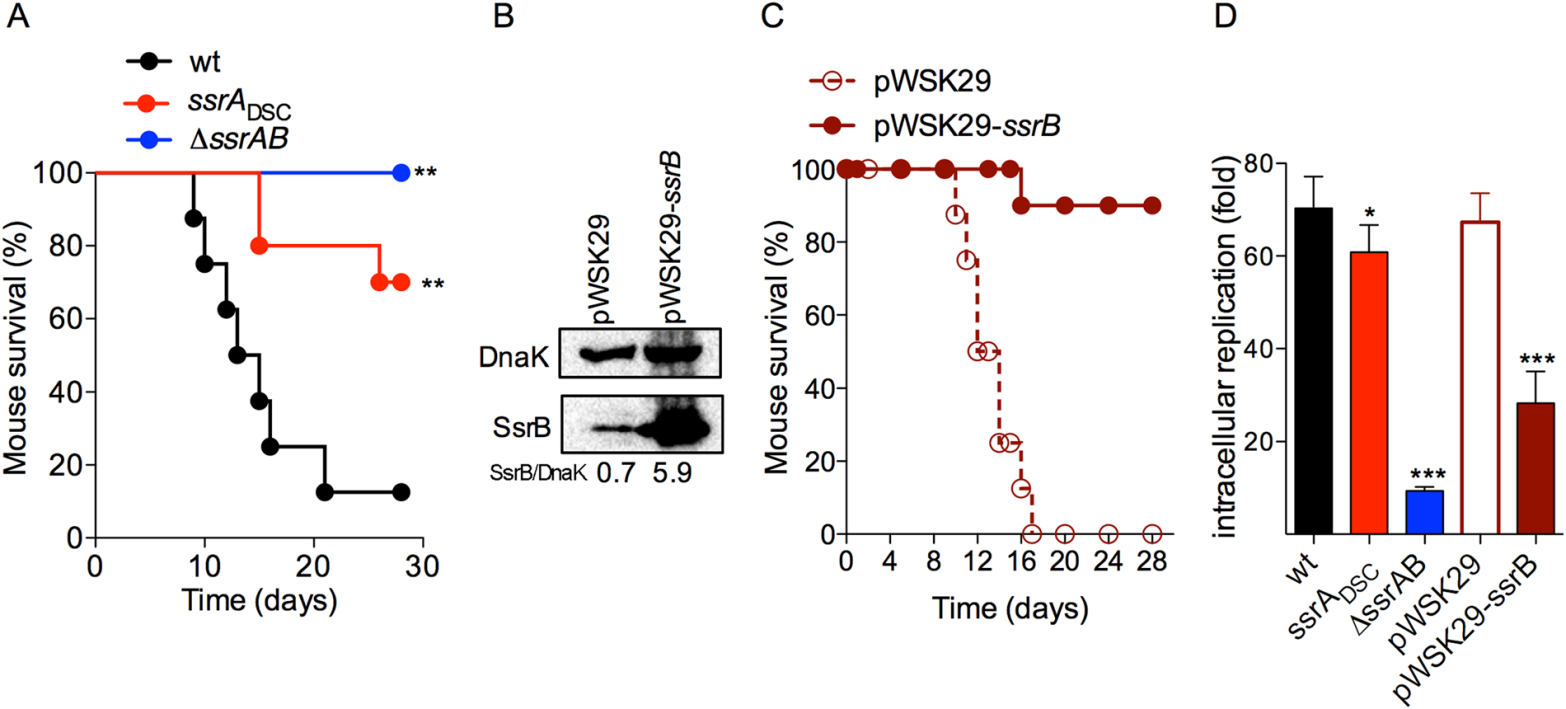
Importance of the AT-rich discriminator region of *ssrA* in *Salmonella* virulence. (**A**) The virulence of wild-type (wt) and mutant *Salmonella* was recorded in a C3H/HeN murine model of oral infection. (**B**) SsrB protein in *Salmonella* expressing the pWSK29 or pWSK29-*ssrB* plasmids. Virulence of pWSK29-*ssrB*^+^ *Salmonella* in a C3H/HeN murine model of acute oral infection. Data in A and C are from 9–10 mice per group. Growth of the indicated *Salmonella* strains in J774 A.1 cells after 20 h of infection (**D**). The data are shown as the mean ± S.D. of 6 biological replicates **p < 0.01, or ***p < 0.001 compared to wild-type controls.

Because some SPI2-dependent phenotypes, such as the one associated with an SsrB C203S variant, were revealed in a C3H/HeN model of oral salmonellosis^24^, we chose this model to test the virulence of *ssrA*_*Dsc*_ *Salmonella*. Moreover, the oral mucosa is the natural route of *Salmonella* infection. We found that *ssrA*_Dsc_ *Salmonella* appear to be as attenuated as Δ*ssrAB* isogenic bacteria when compared to wild-type *Salmonella* (Fig. 5A). These data suggest that the overexpression of SPI2 attenuates *ssrA*_Dsc_ *Salmonella* in a murine model of oral salmonellosis. To test this idea, we evaluated the virulence of a *Salmonella* strain overexpressing the SsrB protein (Fig. 5B). *Salmonella* expressing pWSK29-*ssrB*, not the empty vector, were attenuated when inoculated p.o. into C3H/HeN mice (Fig. 5C). *Salmonella* strains expressing the *ssrA*_DSC_ allele (*p* < 0.05) or the pWSK29-*ssrB* plasmid (*p* < 0.001) grew to lower densities in J774 macrophage-like cells than wild-type controls (Fig. 5D). Collectively, these findings indicate that overexpression of SsrB diminishes *Salmonella* virulence.

## Discussion

Horizontally-acquired and ancestral genes often contain considerable differences in base composition, as exemplified by the SPI2 genes of *Salmonella*^51^. This enteric pathogen has resolved potential difficulties of regulating the SPI2 virulence program by silencing AT-rich promoters with histone-like proteins such as H-NS and YdgT^25,26^. The inhibitory effects of H-NS are counter-silenced by transcription factors such as PhoP and SsrB^19,52^. The AT-rich composition of the discriminator region located between the −10 element and transcription start site can also impose a considerable burden to transcriptional initiation by forming stable, long-lived, open complexes that become saturated with free RNA polymerase^27,28^. Despite the potential burden to transcription, maintenance of an AT-rich discriminator region on *ssrA* suggests that this regulatory element provides a selective advantage to *Salmonella* pathogenesis. Herein, we tested the intriguing possibility that the AT-rich discriminator region serves as a negative regulatory element that is essential for both the appropriate expression of SPI2 gene transcription and *Salmonella* virulence.

In order to investigate whether the *ssrA* AT-rich discriminator region serves as a negative regulatory element, we constructed an *ssrA*_Dsc_ *Salmonella* strain with increased GC-content in the discriminator region. *Salmonella* expressing *ssrA*_Dsc_ overexpressed *ssrA* and *ssrB* genes, which led to enhanced expression of SsrB protein and the downstream *ssaG* gene. The amount of SsrB protein recorded in *ssrA*_Dsc_ *Salmonella* was dramatically reduced when combined with Δ*relA* Δ*spoT* mutations. These findings demonstrate that (p)ppGpp controls the *ssrA* discriminator region and that preservation of an AT-rich discriminator region places control of SPI2 gene transcription under the stimulatory effects of (p)ppGpp. The conversion of the discriminator from GTTTTTA to GTCCCTA may have also affected the repression of *ssrA* locus via the nucleoid proteins H-NS and YdgT. However, the increases in *ssrB* mRNA and SsrB protein noted in the *ssrA*_*Dsc*_ *Salmonella* strain were significantly suppressed when the GC-rich discriminator region was express in Δ*relA* Δ*spoT Salmonella*. These findings strongly argue that the derepression of *ssrB* expression seen in the GC-rich *ssrA*_*Dsc*_ *Salmonella* is dependent on (p)ppGpp rather than through the relief of HN-S, YdgT or StpA binding.

Although appropriate SPI2 expression enables the intracellular replication of *Salmonella*, misregulation of SPI2 transcription seems to diminish the virulence potential of this enteropathogen in a murine model of infection and in macrophage-like J774 cells (herein and^23–26^). The attenuation of *Salmonella* overexpressing SPI-2 genes is analogous to attenuation of *Salmonella* expressing a constitutively active PhoP allele^53^. At present, we don’t know why the overexpression of SsrB attenuates *Salmonella*. In addition to activating SPI-2 gene transcription, SsrB activates the expression of ancestral genes^54^. The overexpression of ancestral or horizontally-acquired genes may be detrimental to *Salmonella* pathogenesis. Together, our investigations emphasize the importance that the repression associated with the AT-rich *ssrA* discriminator region plays in *Salmonella* pathogenesis.

Our investigations indicate that Δ*relA* Δ*spoT Salmonella* are about 100-fold more attenuated than a Δ*ssrB* mutant strain, suggesting roles for (p)ppGpp that are independent of SPI2 gene transcription. This result might reflect the fact that (p)ppGpp regulates 34% of coding RNA transcripts, including SPI1-dependent invasion genes and adaptive stress response programs^38,46,55,56^. Important ways by which this alarmone may contribute to *Salmonella* virulence independently of SPI2 activation include the stringent response to nutritional limitation, alternative sigma factor utilization, mRNA stability, and modulation of translation^34,45,48,55–57^. Nonetheless, the (p)ppGpp-dependent activation of SPI2 transcription might play a sizable role in *Salmonella* pathogenesis as suggested by the fact that the Δ*relA* Δ*spoT Salmonella* is 100-fold more attenuated than Δ*ssrB* controls but over 10^6^-fold more attenuated than wild-type *Salmonella*. A Δ*relA* Δ*spoT Salmonella* strain does not express *ssrAB* mRNA or SsrB protein, indicating (p)ppGpp is fundamental to transcriptional activation of *ssrAB*. Our *in vitro* and *in vivo* transcriptional analyses demonstrate that *ssrAB* is activated through the regulatory effects of (p)ppGpp on the AT-rich *ssrA* discriminator region.

DksA has also been shown to affect open complex stability and often works synergistically with (p)ppGpp^29,31^. It would have been reasonable to predict similar mechanisms in the regulation of SPI2 transcription for both (p)ppGpp and DksA. However, in contrast to (p)ppGpp, our data suggest that DksA does not regulate *ssrAB* transcription. Our biochemical analyses indicate that the amount of SsrB protein, not *ssrB* mRNA, is highly reduced in Δ*dksA Salmonella*, suggesting that DksA regulates *ssrB* expression post-transcriptionally. Transcriptional control of a small RNA could mediate the DksA-dependent post-transcriptional activation of *ssrB*. For example, DksA regulates σ^S^ post-transcriptionally via the small RNA DsrA^58^. Further investigations are needed to elucidate whether DsrA or a small RNA contribute to the DksA-dependent activation of *ssrB*.

Because DksA regulates approximately 10% of the *Salmonella* transcriptome^45^, we were surprised by the remarkable degree of co-dependency between DksA and the SPI2 master regulator SsrB in *Salmonella* pathogenesis. DksA has also been shown to play a major role in the antioxidant and antinitrosative defenses of *Salmonella*^40–43^. By regulating the expression of gene products of central metabolism, cysteine and glutathione biosynthesis, and iron and redox homeostasis, DksA promotes resistance to oxidative and nitrosative stress^39–42^. Our findings herein raise the possibility that the antioxidant and antinitrosative defenses associated with DksA *Salmonella* are not limited to the regulation of NADPH/NADP^+^ and GSH/GSSG redox homeostasis^40–42^. Given the effects that the SPI2 type III secretion system has on vesicular trafficking of NADPH oxidase and iNOS hemoproteins^6,7,10^, it is possible that the regulation of SPI2 gene transcription is a sizable component by which DksA promotes antioxidant and antinitrosative defenses of intracellular *Salmonella*.

Our investigations shed light into the molecular mechanisms by which the stringent response regulators DksA and (p)ppGpp activate the expression of bacterial virulence programs. The stringent response regulators control intracellular spread of *Shigella flexneri*^59^, motility of *Pseudomonas putida*^60^, adherence and virulence of *Haemophilus ducreyi*^61^, and avoidance of lysosomes by *Legionella pneumophila*^62^. DksA and/or (p)ppGpp also regulate the transcription of genes encoding type III secretion systems of *Bordetella pertussis*, *Erwinia amylovora, L. pneumophila*, and *Pseudomonas syringae*^62–65^. In *Salmonella*, DksA activates motility^66^ as well as SPI-1 and SPI-2 type III secretion systems^38^. In most cases, the mechanisms by which these virulence programs are regulated remain unknown. Employing the broadly conserved stringent response regulator (p)ppGpp to overcome the inhibitory barrier imposed by the AT-rich discriminator region of horizontally-acquired pathogenicity islands provides new insights into the regulation of virulence programs in pathogenic bacteria.

### Experimental Procedures

#### Ethics Statement

All methods and experimental procedures were carried out in accordance to protocols approved by the University of Colorado School of Medicine (UCSOM) Institutional Biosafety Committee, authorization number 01–028. Mouse experiments were performed at Animal Care Facility of the UCSOM in accordance to the guidelines established by the UCSOM Institutional Animal Care and Use Committee (IACUC) protocol # 56413(07)1E.

#### Bacterial strains and growth conditions

*Salmonella enterica* serovar Typhimurium strain 14028 s (ATCC, Manassas, VA) and derivative strains are described in Table S1. A 1916-bp DNA fragment, including a 352-bp of the promoter region of the *ssrB* gene, was amplified by PCR from genomic DNA of strain AV07104. The PCR product was directionally cloned into EcoRI/PstI sites of pWSK29, generating the pWSK29-*ssrB* 3 × -FLAG plasmid. *E. coli* strain DH5α (ATCC) was used in molecular cloning. Mutations and plasmids were confirmed by sequencing. Unless specified, bacteria were grown in Luria-Bertani (LB) broth at 37 °C with continuous shaking. When applicable, 20 μg/mL chloramphenicol, 100 μg/mL penicillin, 100 μg/mL ampicillin, 100 μg/mL streptomycin, 20 μg/mL tetracycline, or 50 μg/mL kanamycin were added to the cultures.

#### Construction of *ssrA*_Dsc_*Salmonella*

Segments of a 6.1-kb DNA fragment containing the *ssrAB* operon and a chloramphenicol resistant cassette were amplified from *Salmonella ssrB-*3xFLAG genomic DNA by PCR using the primers described in Table S3 and Figure S5A. PCR products were digested and ligated into pBluescript SK(+) to generate pSK-*ssrAB-*3xFLAG::cm. To introduce the discriminator mutations into the *ssrA* promoter, primer *ssrA*5-F and *ssrA*_Dsc_-R containing the mutations in the discriminator region were used to generate part *ssrA*_Dsc_-P1. The 5′ end of *ssrA* was amplified using primers *ssrA*_Dsc_-F and *ssrA*4-R to generate part *ssrA*_Dsc_-P2. The two *ssrA* segments, *ssrA*_Dsc_-P1 and *ssrA*_Dsc_-P2, were stitched together by PCR elongation. This fragment was ligated into pSK-*ssrAB-*3xFLAG::cm after digestion with *EcoR*I and *Nde*I. The 6.1-kb DNA fragment was digested with *Eco*RI and *Sac*I out of pSK-*ssrAB-*3xFLAG::cm and introduced into Δ*ssrAB*::FRT *Salmonella* by allelic replacement.

#### Allelic replacement

*Salmonella* strains generated in this study followed the method previously described by Datsenko and Wanner^67^ (Table S1). To generate *Salmonella* mutant strains, the plasmids pKD13 and pSK::cm containing a flippase recognition target (FRT)-flanked chloramphenicol cassette was used as a template to generate amplicons with 60-base-pair long primers containing 40-base-pair regions of homology to the gene locus. *Salmonella* strains containing the plasmid pTP223, which expresses the λ Red recombinase from an isopropyl β-D-1-thiogalactopyranoside (IPTG) inducible promoter, were grown in LB broth containing 20 μg/mL tetracycline for 16 h at 37 °C in a shaker incubator. Cells were subcultured 1:100 in LB broth containing 20 μg/mL tetracycline and 1 mg/mL IPTG. Cells were grown for 3 h in a shaker incubator followed by incubation on ice for 30 min. Cells were washed 3-times with 10% glycerol. Approximately 100 ng of DNA were electroporated into bacterial strains using an ECM 399 Exponential Decay Wave Electroporation System (BTX Harvard Apparatus Inc., Holliston, Ma) at 1800 volts for 5 milliseconds. Chromosomal genes were replaced by phage λ Red homologous recombination of electroporated PCR products^67^. Translational fusions containing the promoters of SPI2 genes and *lacZY*^68^ or luciferase^69^ reporter genes were transduced into Δ*dksA* and Δ*relA* Δ*spoT Salmonella* using P22 phage. The strain Δ*relA* Δ*spoT put*::*spoT* was generated by amplifying the genetic locus encompassing *spoT* with *spoT* pSK primers (Table S3) and cloning into pSK::Cm by digestion with ApaI and XhoI. The construct was amplified with *put*::*spoT* primers (Table S3) and recombined into the *Salmonella put* site by allelic replacement

#### Intracellular replication of *Salmonella*

J774A.1 macrophage-like cells (ATCC) were grown in RPMI^+^ medium [RPMI 1640 medium supplemented with 10% heat-inactivated fetal bovine serum (GE Healthcare, Little Chalfont, Buckinghamshire, United Kingdom), 15 mM Hepes, 2 mM L-glutamine, 1 mM sodium pyruvate (Sigma-Aldrich, St. Louis, MO)] at 37 °C in a 5% CO_2_ incubator. J774A.1 cells were infected with stationary phase *Salmonella* that had been grown in LB broth for 20 h at 37 °C in a shaker incubator. Selected groups of macrophages were treated with 200 U/ml IFNγ 20 h before *Salmonella* infection, and where specified, some of the cultures were treated with 960 μM of the iNOS inhibitor L-NIL (Cayman Chemical, Ann Arbor, MI) at the time of infection. J774A.1 cells were infected with stationary phase *Salmonella* at an MOI of 2. Cells were then incubated in RPMI^+^ medium containing 10 μg/ml gentamicin. At 2 h and 18 h post infection, cells were lysed with 0.25% deoxycholic acid and intracellular *Salmonella* were enumerated by dilution plating on LB agar.

#### Quantification of intracellular *sifA::luc* expression

J774A.1 macrophage-like cells were infected at an MOI of 20 with *sifA::luc*-expressing *Salmonella* grown to stationary phase in LB broth for 20 h at 37 °C in a shaker incubator. Extracellular bacteria were removed from the monolayers 25 min after challenge by washing with pre-warmed RPMI^+^ medium containing 50 μg/ml gentamicin. At 8 h post infection, the macrophages were treated with lysis buffer (Promega, Madison, WI) containing 5 mg/mL lysozyme. In parallel, selected macrophages were lysed with 0.25% deoxycholic acid and intracellular bacteria were enumerated on LB agar. Gene expression was measured by following luciferase activity according to the instructions provided by the One-Glo luciferase kit (Promega). Luciferase activity was measured by a Glomax multi-detection system after 5 sec integration in a Lumistar chemiluminometer (Promega). The amount of *sifA::luc* expression is represented as relative light units (RLU) per colony forming unit (CFU).

#### SPI2 induction

*Salmonella* SPI2 induction was performed as previously described^26,68^. *Salmonella* strains grown in LB broth for 16 h at 37 °C in a shaker incubator were subcultured 1:100 in N9 medium [5 mM KCl, 7.5 mM (NH_4_)_2_SO_4_, 0.5 mM K_2_SO_4_, 1 mM KH_2_PO_4_, 38 mM glycerol, 0.1% casamino acids and 100 mM Tris-HCl], pH 7.6 supplemented with 10 mM MgCl_2_ until they reached an OD_600_ of 0.5. The specimens were washed 3 times with 8 μM MgCl_2_ N9 medium, pH 5.8, and then diluted to an OD_600_ of 0.25 in 8 μM MgCl_2_ N9 medium, pH 5.8. After 3 h, cells were pelleted for quantification of SPI2 expression. Alternatively, SPI2 gene expression was induced as *Salmonella* entered into stationary phase in LB broth as previously described^34,38^. Briefly, *Salmonella* grown in LB broth for 16 h at 37 °C in a shaker incubator were subcultured 1:100 into fresh LB broth and grown for 2.5 h or 5 h at 37 °C in a shaker incubator. Independently, SPI2 gene expression was measured in *Salmonella* grown to OD_600_ of 0.5 in M9 minimum medium (7 mg/ml Na_2_HPO_4_, 3 mg/ml KH_2_PO_4_, 0.5 mg/ml NaCI, 1 mg/ml NH_4_Cl, 5 μg/ml thiamine, 0.12 mg/ml MgSO_4_, 0.015 mg/ml CaCl_2_) containing 2 mg/ml glucose and 100 μg/ml casamino acids. Selected samples grown in M9 medium were treated for 30 min with 0.4 mg/mL serine hydroxamate (SHX) previously demonstrated to induce (p)ppGpp accumulation^48^.

#### Quantification of *ssrA* and *ssrB* transcripts by real-time qPCR

*Salmonella* cultures growth in 8 μM MgCl_2_ N9 medium for 3 h were mixed 1:5 (v/v) with an ice-cold solution containing 5% phenol and 95% ethanol. The specimens were placed on ice for 20 min for RNA stabilization. Isolation of bacterial RNA, synthesis of cDNA, and qRT-PCR was performed as previously described^41^. Briefly, RNA was purified using the high pure RNA isolation kit (Roche) according to the instructions provided by the manufacturer. One microgram of total RNA was used to generate cDNA in reactions that contained 100 U MMLV reverse transcriptase (Promega), 0.45 μM N6 random hexamer primers (ThermoFisher Scientific), and 20 U RNAsin Plus RNase inhibitor (Promega). Reverse transcription was performed for 1 h at 42 °C. The primers and probes used for the qRT-PCR are listed in Table  S4. Reactions prepared using TaqMan Gene Expression Master Mix (ThermoFisher Scientific) were incubated for 2 min at 50 °C, followed by 10 min at 95 °C, 40 cycles for 15 sec at 95 °C, and 57 °C for 1 min. Data are expressed as relative expression over the *rpoD* housekeeping gene copy number.

#### *ssrA in vitro* transcription and quantitative RT-PCR

To measure *ssrA in vitro* transcription, we combined *in vitro* transcription reactions with non-radioactive qPCR analysis^49,50^. Briefly, 5 nM pTIM-*ssrA* plasmid was mixed with increasing concentrations of ppGpp (Trilink) in reaction buffer (40 mM HEPES, pH 7.4, 2 mM MgCl_2_, 60 mM potassium glutamate, 0.05% NP-40, 200 μM ATP, 200 μM GTP, 200 μM CTP, 200 μM UTP, and 1 mM DTT). Upon addition of 5 nM *E. coli* RNA polymerase σ^70^ holoenzyme (NEB, Ipswich, MA) to a 10 μl reaction mixture, the *in vitro* transcription reaction was carried out at 37 °C for 10 min, and then terminated at 70 °C for 10 min. DNA-free DNA Removal kit (ThermoFisher) removed template DNA and DNaseI (ThermoFisher). The resulting materials were used as templates to generate cDNA with 100 U M-MLV reverse transcriptase (Promega), 0.45 μM N6 random hexamer primers (ThermoFisher), and 20 U RNase inhibitor (Promega). The amount of cDNA synthesized for 1 h at 42 °C was quantified by real-time PCR (qRT-PCR) using the primers and probe described in Table S4. The *ssrA* specific transcripts were normalized to the standard curve generated with known *ssrA* gene copy concentrations.

#### Western blotting

*Salmonella* expressing *ssrB* with a C-terminal FLAG epitope^24^ were cultured in SPI2-inducing 8 μM MgCl_2_ N9 medium as described above. After 3 h, cultures were centrifuged at 10,000 g for 5 min and bacterial pellets were stored at −80 °C. Samples were lysed by sonication in 125 mM NaCl Tris buffer, pH 7.0. Cellular debris was pelleted upon centrifugation at 16,000 g for 5 min. The protein concentration was determined with a Pierce 660 nm Protein Assay Reagent (ThermoFisher Scientific). Total soluble proteins (500 ng) resolved in 12% (v/v) SDS-PAGE gels were transferred electrophoretically to nitrocellulose membranes. The membranes were blocked with 5% milk, and immunoblotted with a 1:500 dilution of mouse IgG1 anti-FLAG M2 (Sigma-Aldrich) or a 1:2500 dilution of mouse IgG anti-DnaK (MBL International Corporation, Woburn, MA) monoclonal antibodies. The membranes were probed with 1:5,000 of HRP-conjugated sheep anti-mouse IgG secondary antibody (GE Healthcare). The signals in the membranes, developed with an Amersham ECL Prime Western Blotting Detection Reagent (GE Healthcare), were visualized with a Molecular Imager ChemiDoc XRS + system (Bio-Rad).

#### Competitive index assay

The relative contribution of *ssrB, dksA*, and (p)ppGpp to *Salmonella* virulence was quantified by recording the competitive index of mutant and wild-type isogenic controls. Briefly, C57BL/6 J (The Jackson Laboratory, Mount Desert Island, ME) mice bred in our animal facility according to institutional guidelines were infected i.p. with about 10^2^ or 10^5^ CFU of *Salmonella* grown to stationary phase in LB broth for 20 h at 37 °C in a shaker incubator. The bacteria used for inoculation were prepared in PBS. Spleens and livers collected 3 days after infection were macerated in PBS, and the amount of *Salmonella* present in the tissues was enumerated by dilution replica-plating on LB agar containing the appropriate antibiotics. The competitive index was calculated as:

(3) (strain 1/strain 2)_output_/(strain 1/strain 2)_input_.

#### Mouse survival

The virulence of *ssrA*_Dsc_-expressing *Salmonella* was investigated in C3H/HeN mice (The Jackson Laboratory) that were bred in the CU Anschutz animal facility. Briefly, C3H/HeN mice were infected orally with 10^7^ CFU of the indicated *Salmonella* strains that had been grown in LB broth for 20 h at 37 °C in a shaker incubator. The bacteria used for inoculation were prepared in PBS. Mice survival was monitored for 28 days. The data are from 10 mice.

#### Statistical Analysis

Statistical analysis and graphing were performed using GraphPad Prism 4.0 software. Determination of statistical significance between two comparisons was achieved using an unpaired *t*-test. Determination of statistical significance between multiple comparisons was done using a one-way analysis of variance (ANOVA) followed by Bonferroni or Dunnett’s multiple comparison post-test with respective isogenic strain as control. To determine statistical significance for competitive indexes, one-way ANOVA or Mann-Whitney tests were used. Statistical significance for C3H/HeN mice survival curves was determined using log-rank test, comparing mutant *Salmonella* strain to wild-type controls.

## Electronic supplementary material

Supplementary information
