# Supplementary material for: Guanosine tetraphosphate relieves the negative regulation of Salmonella pathogenicity island-2 gene transcription exerted by the AT-rich ssrA discriminator region
Source: Sci Rep. 2018 Jun 21;8:9465. doi: 10.1038/s41598-018-27780-9 (PMC6013443; doi:10.1038/s41598-018-27780-9)
Supplement: Supplementary file 1 — Supplementary information [file 41598_2018_27780_MOESM1_ESM.pdf]

Guanosine tetraphosphate relieves the negative regulation of *Salmonella* pathogenicity island-2 gene transcription exerted by the AT-rich *ssrA* discriminator region

Timothy Tapscott, Ju-Sim Kim, Matthew A. Crawford, Liam Fitzsimmons, Lin Liu, Jessica Jones-Carson, and Andrés Vázquez-Torres

Supplementary Information

Supplementary Experimental Procedures

NO_2^- determination. $^{\bullet}NO$ synthesis by interferon-gamma (IFNγ)-stimulated J774A.1 cells was determined by measuring nitrite (NO_2^-) generated by the reaction of nitric oxide with oxygen. NO_2^- released into the culture supernatants by the macrophages 18 h after infection was measured after the addition of an equal volume of Griess reagent (0.5% sulfanilamide and 0.05% N-1-naphthylethylenediamide hydrochloride in 2.5% phosphoric acid). The resulting change in color was read at 550 nm in a Versa Max spectrophotometer (Molecular Devices, Sunnyvale, CA). The NO_2^- concentration was determined from a standard curve prepared with $NaNO_2$.

Quantification of β-galactosidase expression. *Salmonella* strains expressing the *lacZY* translational fusions were lysed with chloroform in 980 μL of Z-buffer (0.06 M Na_2HPO_4, 0.04 M NaH_2PO_4, 0.01 M KCl, 0.001 M $MgSO_4$ and 0.05 M β-mercaptoethanol) containing 0.002% SDS (w/v). Samples were equilibrated at 30°C prior to the addition of 200 μL of 4 mg/mL ortho-nitrophenyl-β-galactoside (Sigma-Aldrich). The reactions were terminated by the addition of 500 μL of 1 M Na_2CO_3. β-galactosidase activity was measured with a Versa Max spectrophotometer

(Molecular Devices, Sunnyvale, CA) at 420 nm and 550 nm. Data are expressed in Miller units according to the equation:

$$(2)\ 1{,}000 \times [(OD_{420} - 1.75 \times OD_{550})]/(T(\text{min}) \times V(\text{ml}) \times OD_{600}).$$

Table S1.
Bacterial
Strains

**Strain**	**Genotype**	**Source**
S. Typhimurium strain 14028s	Wild-type	ATCC
E. coli DH5α	F-φ80*lac*ZΔM15 Δ(*lac*ZYA-*arg*F) U169 *rec*A1 *end*A1 *hsd*R17 (rK^-, mK^+) *pho*A *sup*E44 λ– *thi*-1 *gyr*A96 *rel*A1	ATCC
AV07270	Δ*dksA*::cm	[1]
AV08140	Δ*relA*::FRT Δ*spoT*::FRT	[2]
AV00203	Wild-type *sifA*::*lacZY*-km	[3]
AV15023	Δ*dksA*::cm *sifA*::*lacZY*-km	This study
AV15024	Δ*relA*::FRT Δ*spoT*::FRT *sifA::lacZY*-km	This study
AV00204	Wild-type *srfJ*::*lacZY*-km	[3]
AV15005	Δ*dksA*::cm *srfJ*::*lacZY*-km	This study
AV15006	Δ*relA*::FRT Δ*spoT*::FRT *srfJ::lacZY*-km	This study
AV00205	Wild-type *sspH2*::*lacZY*-km	[3]
AV15008	Δ*dksA*::cm *sspH2*::*lacZY*-km	This study
AV15009	Δ*relA*::FRT Δ*spoT*::FRT *sspH2*::*lacZY*-km	This study
AV00207	Wild-type *spiC*::*lacZY*-km	[3]
AV15011	Δ*dksA*::cm *spiC*::*lacZY*-km	This study
AV15012	Δ*relA*::FRT Δ*spoT*::FRT *spiC*::*lacZY*-km	This study
AV00206	Wild-type *sseE*::*lacZY*-km	[3]
AV15017	Δ*dksA*::cm *sseE*::*lacZY*-km	This study
AV15018	Δ*relA*::FRT Δ*spoT*::FRT *sseE*::*lacZY*-km	This study
AV15013	Wild-type pQF50-*ssaG*	This study
AV15014	Δ*dksA*::cm pQF50-*ssaG*	This study
AV15015	Δ*relA*::FRT Δ*spoT*::FRT pQF50-*ssaG*	This study
AV15019	Wild-type pQF50-*sseA*	This study
AV15020	Δ*dksA*::cm pQF50-*sseA*	This study
AV15021	Δ*relA*::FRT Δ*spoT*::FRT pQF50-*sseA*	This study
AV11276	Wild-type *sifA*::*luc*	[4]
AV15027	Δ*dksA::*cm *sifA*::*luc*	This study
AV15028	Δ*relA* Δ*spoT sifA*::*luc*	This study

AV14025	Wild-type Str^{R}	This study
AV13150	Δ*dksA*::cm Str^{R}	This study
AV13146	Δ*ssrB*::km Str^{R}	This study
AV13149	Δ*ssrB*::km Δ*dksA*::cm	This study
AV14025	Δ*relA*::FRT Δ*spoT*::cm Str^{R}	This study
AV14043	Δ*relA*::FRT Δ*spoT*::cm Δ*ssrB*::km	This study
AV11228	Wild-type *ssrB*-FLAG	This study
AV15189	Δ*dksA*::cm *ssrB*-FLAG	This study
AV15190	Δ*relA*::FRT Δ*spoT*::FRT *ssrB*-FLAG	This study
AV15162	Δ*ssrAB::*FRT	This study
AV07104	Wild-type *ssrB*-3xFLAG	[5]
AV15202	*ssrA*$_{Dsc}$ *ssrB*-3xFLAG	This study
AV10369	Δ*dksA*::FRT *put*::*dksA*	[6]
AV16176	Δ*relA*::cm Δ*spoT*::km *put*::*spoT*::FRT	This study
AV18094	Wild-type *Salmonella* with pWSK29	This study
AV18096	Wild-type *Salmonella* with pWSK29-ssrB-3xFLAG	This study

Table S2.

Plasmids

Plasmid	Relevant Genotype	Source
pQF50	*bla lacZ*	[7]
pQF50-*ssaG*	*bla* P*ssaG*(-276/+35) *lacZ*	This study
pQF50-*spiC*	*bla* P*spiC*(-195/+166) *lacZ*	This study
pGEX6P1	*bla* P*lacZ* GST	GE Healthcare
pIDTSmart amp	*bla*	IDT
pTIM	*bla* pIDTSmart amp *rrnB* & *rpoC* term	This study
pTIM-*ssrA*	*bla* pIDTSmart amp P*ssrA(-258/+1202)*	This study
pSK::cm	*bla* FRT *cat* FRT pUC *ori* f1 *lacZ*α	This study
pKD13::km	*bla* FRT *ahp* FRT *oriR6K*	[8]
pTP223	P*lac*-gam-bet-exo	[9]
pWSK29	*bla lacZ*α T7/T3 *ori* f1 pSC101ori	[10]
pWSK29-ssrB-3xFLAG	*ssrB-3xFLAG* (-352 - +1564)	This study

Table S3. Primers

**Gene**		**Primer Sequence**
pTIM-*ssrA*	F:	CGGAATTCCGCCAGCATGAATCCCTCCTC
	R:	GGGGTACCCCTTGTGCTGGTAAACGTGTGC
Δ*ssrAB*	F:	ACTTACAATTTGAAAAATTATTTATTAAATAAACTGTTACGTGTAGGCTG GAGCTGCTTCG
	R:	CGAAGCGACCACGTTGCGCCACTGGGCAAGCTGTTTTTCTGCATTCC GGGGATCCGTCGAC
ssrA$_{\text{Dsc}}$	F:	CATCGCCATCTTATTAAAAAGTAATTG
	R:	CAATTACTTTTTAATAAGATGGCGATGTAGGCACATCGTAACAGTTTAT TTAATAAATAATTT
*ssrA*5	F:	GAATTCACATTTATTTCGACTATAC
*ssrA*3	R:	GCTGCCCTCGCGAAAATTAAG
*ssrA*4	R:	GACAAAAGTACGTAATGACAG
*ssrB*4	F:	GAATTCAGAGCTACAGGAGCAGGATC
orf242-1	R:	CTGCAGCGCCTATAGTGTGATAAC
orf242-2	F:	ACTAGTTAGATTTCTTCCCCTCATTC
orf242-3	R:	GAGCTCATCAAAGCGTACCGTGGCGCCA
cmP2	F:	CTGCAGCATGGTCCATATGAATATCC
	R:	ACTAGTGTGTAGGCTGGAGCTGCTTC
Δ*relA*::FRT Δ*spoT*::FRT *put*::*spoT*		
spoT pSK	F:	TAGGGCCCAGGTATAGCGCTTTAGTGAATAAAAACCG
	R:	GCCTCGAGCTAGTTTCGGTTACGGGTGA
put::*spoT*	F:	TAGCGATGGGAGAGAGGACACGTTAATTATTCCATTTTAAAGGTATA GCGCTTTAGTGAATAAAAACCG
	R:	TACTGCGGGTATTAATGCTGAAAACATCCATAACCCATTGGTGTAGG CTGGAGCTGCTTC
ssrB-3xFLAG	F:	GAATTCAGAGCTACAGGAGCAGGATC (underlined, EcoRI site)
	R:	CTGCAGCGCCTATAGTGTGATAAC (underlined, PstI site)

Table S4. Primers and probes for qPCR and *in vitro* transcription

**Gene**		**Primer Sequence**
ssrA[1]	F:	ATATTACGCACAACCTTGCAT
	R:	CCAGTGAGCGATGTAGTAACCA
	Probe:	AAGCCGACGTCATCAACACCA
ssrA[2]	F:	TCATCGACTGGGTTATATATGAAG
	R:	AGATTGAGCAAATTCATAATGCTT
	Probe:	CTTTGGCACTTGATCACTATCGC
ssrB	F:	AGCGGCATTGCAAACAGT
	R:	TACCAATCATGGGATCAGCG
	Probe:	ATCGGGAAGCTATCCTGGCTG
ssaG	F:	TCCCACATGGCGCACCAG
	R:	ATGATTCCACTAAGCATATCCTTGA
	Probe:	AAGCGCAATTTGCCTTACAGCAG

[1]Primer set and probe used for *ssrA* qPCR

[2]Primer set and probe used for *in vitro* transcription

Supplementary Figure Legends

Fig S1. Interactions of *Salmonella* with J774A.1 cells. The amount of nitrite (NO_2^-) generated by macrophages 18 h after *Salmonella* infection was quantified by the Griess reaction (A). J774A.1 cells were stimulated with 200 U/ml IFNγ 24 h prior to infection, or treated with 960 μM of the selective iNOS inhibitor N-iminoethyl-L-lysine (L-NIL) since the time of infection. The data represent the mean ± S.D. from at least 3 biological replicates. *** $p < 0.001$ as compared to untreated control (A). Transcriptional analysis of major SPI2 promoters fused to a promoterless *lacZY* reporter in *Salmonella* (B). Fold induction is the ratio of β-galactosidase enzymatic activity 3 h after culture of *Salmonella* in 8 μM $MgCl_2$ N9 medium over controls grown in 10 mM $MgCl_2$ N9 medium. The data are the mean ± S.D. from 3 biological replicates. *** $p < 0.001$ as compared to wild-type controls.

Fig S2. Competitive index of *Salmonella* strains. Competitive indices of *Salmonella* strains recovered livers of C57BL/6 mice 3 d after infection. Mice were inoculated i.p. with 10^2 (A) or 10^5 (B) CFU of the indicated *Salmonella* strains. No detectable (nd) CFU were isolated for the Δ*relA* Δ*spoT* strain under the experimental conditions used in panel A. Competitive index was determined by the equation $(\text{strain 1/strain 2})_{output}/(\text{strain 1/strain 2})_{input}$. Non-significant (ns), or *$p < 0.05$.

Fig. S3. SsrB protein expression in Δ*dksA* *Salmonella* complemented with a *dksA* allele. SsrB expression was determined by Western blotting in the indicated strains of *Salmonella*. Δ*dksA* *Salmonella* was complemented with the low copy plasmid pWSK29 containing the *dksA* gene. Two independent clones are shown for comparison.

Fig. S4. Map of the pTIM plasmid used for the *in vitro* transcription reactions. pTIM plasmid containing two multiple cloning sites (MCS) and two Rho-independent terminators (A). DNA containing MCS 1 and MCS 2 (blue) and *rrnB* and *rpoC* terminators (underlined) was inserted into pIDTSmart backbone by *in vitro* synthesis (B). The plasmids resulting from cloning gene promoters into MCS 1 were used as templates for *in vitro* transcription reactions.

Fig S5. Cloning strategy for the construction of *Salmonella* $ssrA_{\mathrm{Dsc}}$. The *ssrAB* locus was was cloned into pBluescript SK(+) containing *ssrAB*, *orf242*, and a chloramphenicol resistant cassette, yielding pSK-*ssrAB*-3xFLAG::cm plasmid. Mutations in the discriminator region were introduced by subcloning the *ssrA* promoter with a reverse primer containing the discriminator mutations ($ssrA_{\mathrm{Dsc}}$-R) and the *ssrA5*-F containing an *Eco*RI site. The resulting product generated the $ssrA_{\mathrm{Dsc}}$-P1 fragment. The $ssrA_{\mathrm{Dsc}}$-P1 promoter was stitched by PCR to the fragment $ssrA_{\mathrm{Dsc}}$-P2 containing an *Nde*I site. The $ssrA_{Dsc}$ was reintroduced to pSK-*ssrAB*-3xFLAG::cm by digesting and ligating with *Eco*RI and *Nde*I sites. Western blot analysis of SsrB in wild-type and $ssrA_{Dsc}$ *Salmonella* grown in high and low Mg^{2+} media (B). DnaK was used as internal control. Ratio of SsrB signal / DnaK signal was calculated from densitometry in ImageJ.

Fig S6. Full size Western blots. Blots developed with an Amersham ECL Prime Western Blotting Detection Reagent (GE Healthcare and visualized with a Molecular Imager ChemiDoc XRS+ system (Bio-Rad). Panel A depicts the full blot of the cropped image shown in Fig 3C, and panel B depicts the full blot of the cropped image shown in Fig 4D.

References

1 Henard, C. A., Bourret, T. J., Song, M. & Vazquez-Torres, A. Control of redox balance by the stringent response regulatory protein promotes antioxidant defenses of *Salmonella*. *J Biol Chem*. **285**, 36785-36793 (2010).

2 Henard, C. A. & Vazquez-Torres, A. DksA-dependent resistance of *Salmonella enterica* serovar Typhimurium against the antimicrobial activity of inducible nitric oxide synthase. *Infect Immun*. **80**, 1373-1380 (2012).

3 McCollister, B. D., Bourret, T. J., Gill, R., Jones-Carson, J. & Vázquez-Torres, A. Repression of SPI2 transcription by nitric oxide-producing, IFNγ-activated macrophages promotes maturation of *Salmonella* phagosomes. *J Exp Med*. **202**, 625-635 (2005).

4 Xu, X. & Hensel, M. Systematic Analysis of the SsrAB Virulon of *Salmonella enterica*. *Infect Immun*. **78**, 49-58 (2010).

5 Husain, M. *et al.* Redox sensor SsrB Cys203 enhances *Salmonella* fitness against nitric oxide generated in the host immune response to oral infection. *Proc Natl Acad Sci U S A*. **107**, 14396-14401 (2010).

6 Crawford, M. A. *et al.* Redox-active sensing by bacterial DksA transcription factors is determined by cysteine and zinc content. *mBio*. **7**, e02161-02115 (2016).

7 Jobling, M. G. & Holmes, R. K. Characterization of *hapR*, a positive regulator of the *Vibrio cholerae* HA/protease gene *hap*, and its identification as a functional homologue of the *Vibrio harveyi luxR* gene. *Mol Microbiol*. **26**, 1023-1034 (1997).

8 Datsenko, K. A. & Wanner, B. L. One-step inactivation of chromosomal genes in *Escherichia coli* K-12 using PCR products. *Proc Natl Acad Sci U S A*. **97**, 6640-6645 (2000).

9 Murphy, K. C. Use of bacteriophage λ recombination functions to promote gene replacement in *Escherichia coli*. *J Bacteriol*. **180**, 2063-2071 (1998).

10 Rong Fu, W. & Kushner, S. R. Construction of versatile low-copy-number vectors for cloning, sequencing and gene expression in *Escherichia coli*. *Gene*. **100**, 195-199 (1991).

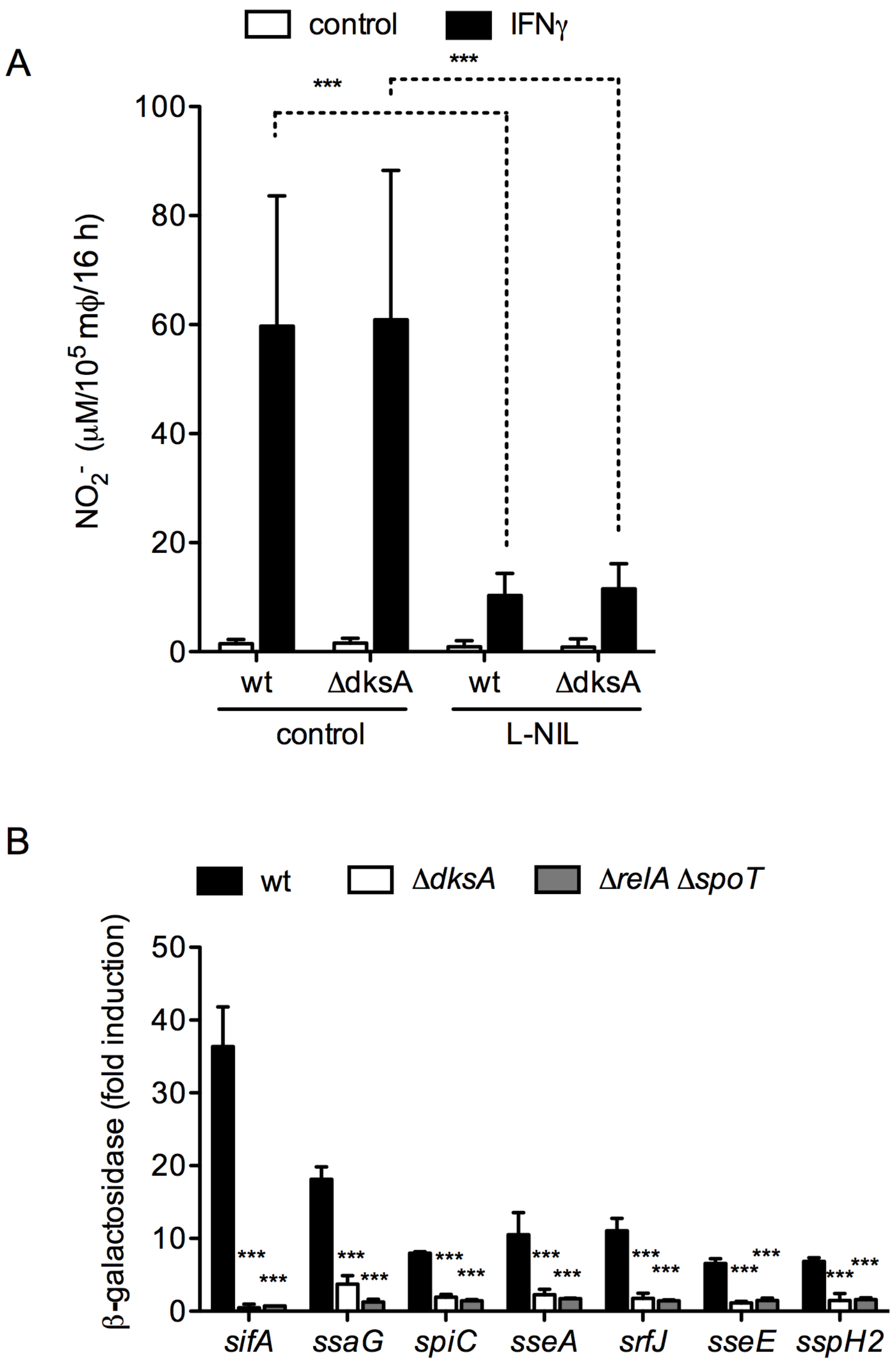
control
IFNγ
A
***
***
100
80
60
40
20
0
NO2- (μM/10^5 mϕ/16 h)
wt
ΔdksA
wt
ΔdksA
control
L-NIL
B
wt
ΔdksA
ΔrelA ΔspoT
50
40
30
20
10
0
β-galactosidase (fold induction)
sifA
ssaG
spiC
sseA
srfJ
sseE
sspH2

Fig. S1

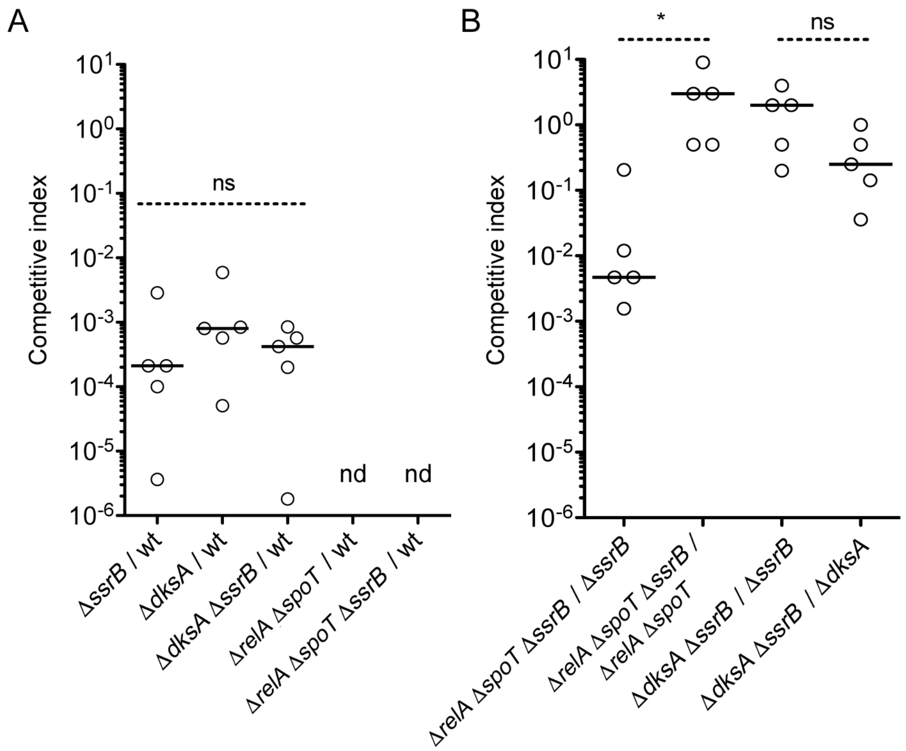
A
B
Competitive index
ns
*
ns
nd
nd
ΔssrB / wt
ΔdksA / wt
ΔdksA ΔssrB / wt
ΔrelA ΔspoT / wt
ΔrelA ΔspoT ΔssrB / wt
ΔrelA ΔspoT ΔssrB / ΔssrB
ΔrelA ΔspoT ΔssrB / ΔrelA ΔspoT
ΔdksA ΔssrB / ΔssrB
ΔdksA ΔssrB / ΔdksA

Fig. S2

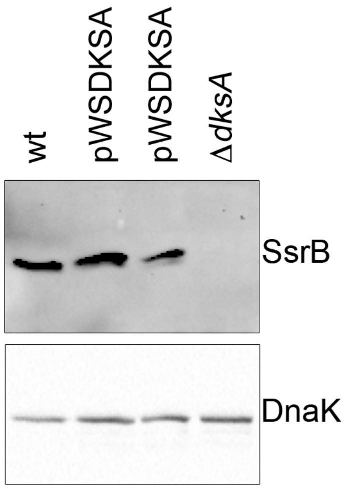

Fig. S3

A

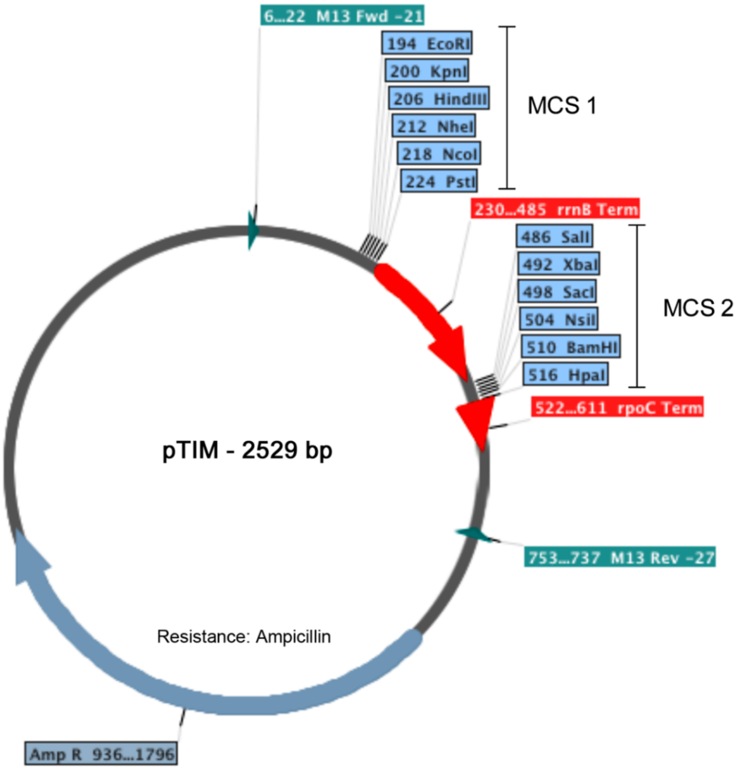

B

```
                MCS 1
 -------------------------------------
                 >HindIII           >PstI
           >Kpn1              >Nco1
     >EcoR1             >NheI
      |     |     |     |     |     |
ATCGAATTCggtaccAAGCTTgctagcCCATGGctgcagGCGAGAGTAGGGAACTGCCAGGCATCAAATAAAACGAAAGG

CTCAGTCGAAAGACTGGGCCTTTCGTTTTATCTGTTGTTTGTCGGTGAACGCTCTCCTGAGTAGGACAAATCCGCCGGGA

      >rrnB Terminator
      |
GCGGATTTGAACGTTGCGAAGCAACGGCCCGGAGGGTGGCGGGCAGGACGCCCGCCATAAACTGCCAGGCATCAAATTAA

                                                                    MCS 2
                                                        -------------------------
                                                                     >SacI
                                                               >XbaI
                                                         >SalI             >NsiI
                                                          |     |     |     |
GCAGAAGGCCATCCTGACGGATGGCCTTTTTGCGTTTCTACAAACTCTTCCTGTCgtcgacTCTAGAgagctcATGCATg

-----------
      >HpaI
 >BamHI                                                 >rpoC Terminator
 |     |                                                |
gatccGTTAACTCTGGGCGGTTCTGATAACGAGTAATCGTTAATCCGCAAATAACGTAAAAACCCGCTTCGGCGGGTTTT

TTTATGGGGGGAGTTTAGGGA
```

Fig. S4

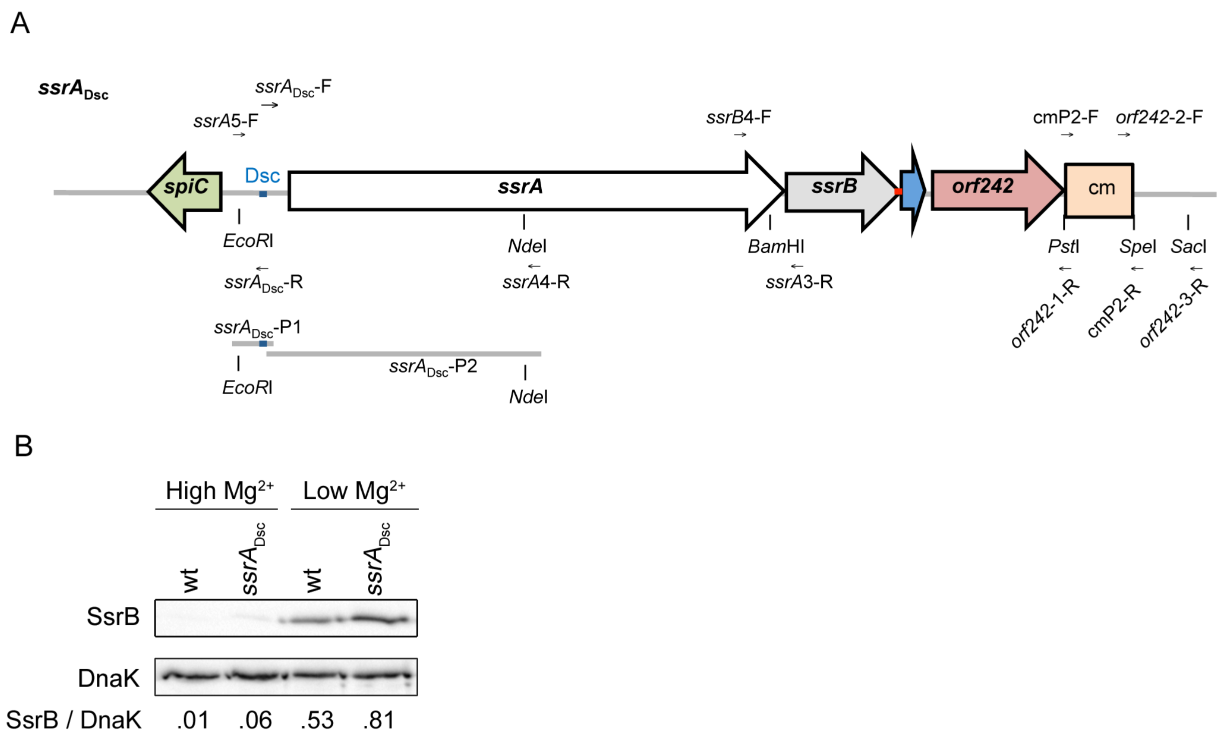
A
ssrA_Dsc
ssrA_Dsc-F
ssrA5-F
ssrB4-F
cmP2-F
orf242-2-F
spiC
Dsc
ssrA
ssrB
orf242
cm
EcoRI
NdeI
BamHI
PstI
SpeI
SacI
ssrA_Dsc-R
ssrA4-R
ssrA3-R
orf242-1-R
cmP2-R
orf242-3-R
ssrA_Dsc-P1
ssrA_Dsc-P2
EcoRI
NdeI
B
High Mg2+
Low Mg2+
wt
ssrA_Dsc
wt
ssrA_Dsc
SsrB
DnaK
SsrB / DnaK
.01
.06
.53
.81

Fig. S5

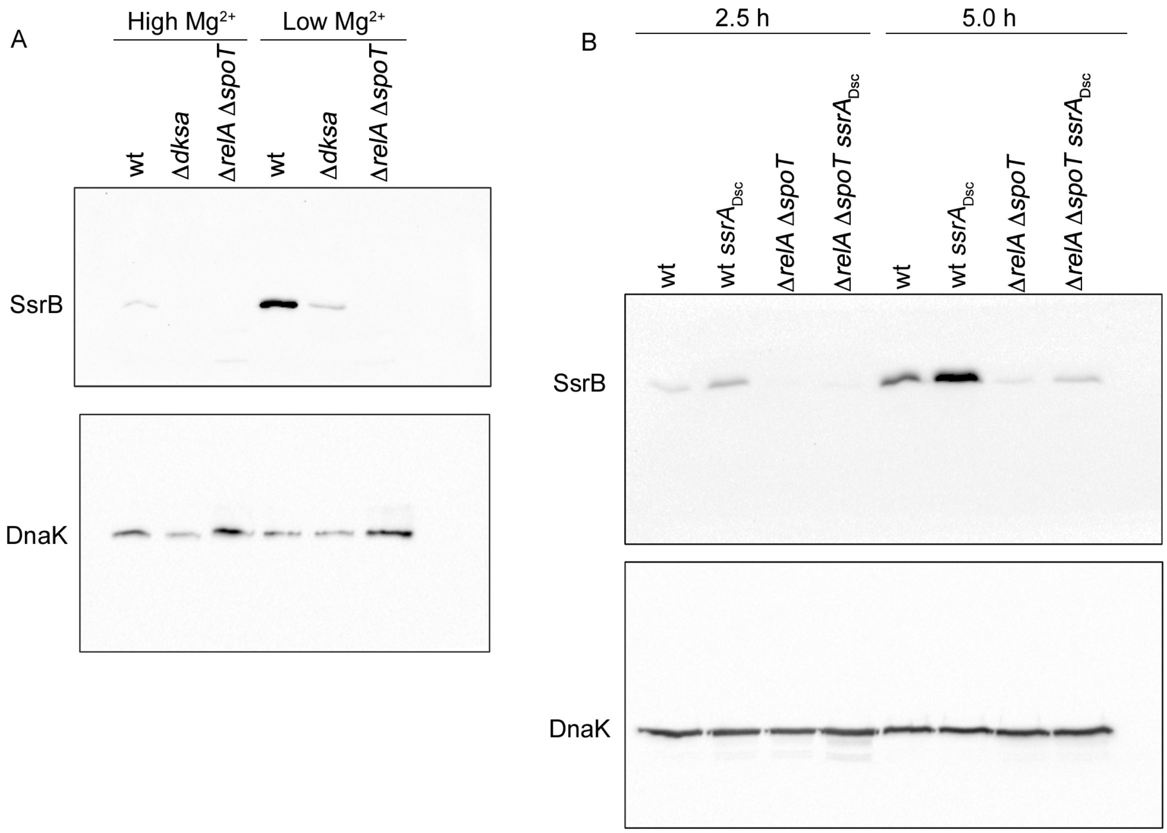
A
High Mg^{2+}
Low Mg^{2+}
wt
Δdksa
ΔrelA ΔspoT
wt
Δdksa
ΔrelA ΔspoT
SsrB
DnaK
B
2.5 h
5.0 h
wt
wt $ssrA_{Dsc}$
ΔrelA ΔspoT
ΔrelA ΔspoT $ssrA_{Dsc}$
wt
wt $ssrA_{Dsc}$
ΔrelA ΔspoT
ΔrelA ΔspoT $ssrA_{Dsc}$
SsrB
DnaK

Fig. S6
